# Artificial intelligence and real-time directional guidance in arrhythmia management

**DOI:** 10.1016/j.hroo.2025.08.025

**Published:** 2025-09-04

**Authors:** Sanjiv M. Narayan

**Affiliations:** 1 Department of Medicine, Stanford University, Palo Alto, California; 2 Cardiovascular Institute, Stanford University, Palo Alto, California; 3 Institute for Computational and Mathematical Engineering, Stanford University, Palo Alto, California; 4 School of Information and Data Sciences, University of California, Berkeley, Berkeley, California

**Keywords:** Atrial fibrillation, Machine learning, Ablation outcome, Phenotyping, Resource utilization

**Key Findings**

▪ A novel artificial intelligence (AI) system is described that provides real-time directional guidance during atrial fibrillation (AF) and adapts to changes in real-time during a case.
▪ This approach points toward candidate ablation targets in AF from any starting point, without the need to map the entire atrium.
▪ This approach demonstrates the potential of AI to simplify catheter ablation for complex arrhythmias and to enhance the ability of electrophysiologists across all levels of expertise.

There is a need to simplify and improve procedural therapy for patients with complex heart rhythm disorders. Anatomically-based procedures can now be performed safely, effectively, and rapidly, such as pulmonary vein (PV) isolation (PVI) or posterior wall isolation for atrial fibrillation (AF),^1^ or ablation for many supraventricular arrhythmias.^2^ However, arrhythmias arising from non-stereotypical locations remain challenging to treat, such as AF with isolated PVs, atypical flutters after ablation, and many ventricular tachycardias. Treating these arrhythmias often requires time-consuming mapping, as well as expert interpretation of complex signals, which have had mixed clinical results.^1^^,^^2^

**Figure 1:**
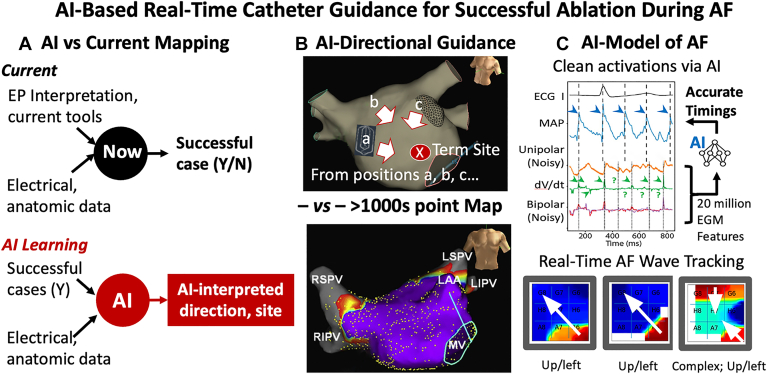
Artificial intelligence (AI)-based active directional guidance. **A:** The AI-paradigm flips current practice—learning from complex data in known cases to provide automated AI-guidance. **B:** AI provides real-time directional guidance from different locations (a, b, c shown). This can be repeated successively after small spatial steps to arrive at a promising ablation site, without creating a time-averaged map of the entire chamber. **C:** The interpretable AI-model is trained to physiological ground truth labels, and estimates real-time wave tracking. AF = atrial fibrillation; ECG = electrocardiogram; EGM = electrogram; EP = electrophysiology; MAP = monophasic action potentials; N = no; Y = yes.

Artificial intelligence (AI) can codify and enhance expert experience by learning from large, labeled datasets,^3^ and has enabled several innovations in electrophysiology.^4^ AI has been used in AF to identify intracardiac electrogram dispersion as ablation targets,^5^ to identify AF sources proposed by the Horn-Schunck algorithm,^6^ and to validate AF drivers relative to ex vivo optical mapping.^7^ In general, there is a need for procedural AI tools that are able to decipher arrhythmia patterns hidden by the chaotic nature of AF. As shown in Figure 1A, novel AI could reverse current approaches by learning how to interpret physiological signals from the outcome of known cases, rather than relying solely on expert reasoning to solve them.

One novel AI-paradigm that could simplify procedural management of complex arrhythmias is real-time directional guidance. This paradigm could enable an AI algorithm to ingest local electrograms at any given catheter position, and in real-time compute the next direction to reach rapid sites, gaps in ablation lines, or other targets. As the catheter moves, the system would update directional guidance within seconds, producing an adaptive trajectory that converges to candidate sites for ablation. This approach could extend PVI, such as guiding a user directly to extra-PV foci if the veins are isolated, without the need to map the entire chamber. During ablation of atypical atrial flutter, such a system could immediately identify subtle rhythm changes and update directions accordingly.

We present a 67-year-old woman with persistent AF despite 3 prior ablations at an outside hospital, including PVI. She had a history of hypertension and mitral valve prolapse, had tried amiodarone which but this was discontinued after increases in liver enzymes, and presented for re-ablation. During AF, a multipolar catheter was placed in the left atrium (Figure 1B). In Figure 1C, the AI model actively filters noise from AF electrograms, isolating physiologically meaningful signals^8^ to provide real-time wave tracking and derive directional guidance.^9^ Figure 1B shows that computed directions pointed to the same site—anterior left atrium—from catheter positions (a), (b), (c), and several additional sites in this patient. The system ultimately identified the site marked “X” where ablation acutely terminated AF. The patient subsequently had no AF at 1-year follow-up. This real-time, AI-based directional approach avoids constructing a map over many minutes that could miss rapid changes in an arrhythmia.

In conclusion, we describe a novel application of AI to streamline analysis of the vast, complex data performed by an electrophysiologist during a procedure, to help guide catheter movement in real-time. With careful design, AI systems trained to validated physiological end points may facilitate transparency, with the potential to simplify procedures, increase success rates, and improve procedural efficiency.
